# Supplementary material for: Verification of Key Target Molecules for Intramuscular Fat Deposition and Screening of SNP Sites in Sheep from Small-Tail Han Sheep Breed and Its Cross with Suffolk
Source: Int J Mol Sci. 2024 Mar 3;25(5):2951. doi: 10.3390/ijms25052951 (PMC10931736; doi:10.3390/ijms25052951)

Supplementary Table S1: Primer Sequence

Gene name	Accession NO.	Sequence 5'-3'	Product Length
PIK3R1	XM_004016920.4	F: CCCACCATGACGAGAAGACT<br>R: GCCCTGTTTACTGCTTTCCC	116 bp
PPARA	XM_027968213.2	F: ATCGAGTGTAGGATCTGCGG<br>R: CGCATCTGTCATACACCAGC	121 bp
GAPDH	NM_001190390.1	F: CCATCTTCCAGGAGCGAGAT<br>R: TGGTCATAAGTCCCTCCACG	137 bp

Supplementary Table S2: Statistical analysis of IMF content

Sample	%Area	Mean	IntDen
STH	2.462	62.819	48014.94
STH	2.731	85.183	31832.84
STH	2.731	85.183	31832.84
STH	1.848	64.447	26144.59
STH	1.798	61.889	25955.27
STH	1.794	61.752	25867.58
SFK×STH	23.272	103.331	194767.3
SFK×STH	23.342	105.449	179256.5
SFK×STH	23.161	100.452	205817.5
SFK×STH	16.24	108.651	91621.73
SFK×STH	15.76	94.508	154686
SFK×STH	15.402	88.245	164132.4

Supplementary Figure S1: DNA Electrophoresis Detection Results of Sheep Blood Group

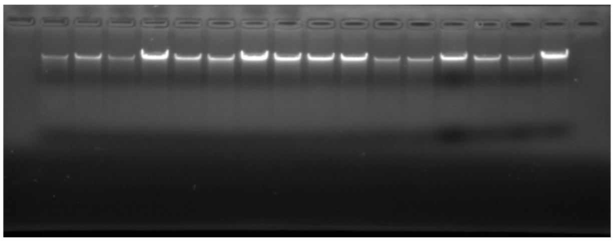

Supplementary Figure S2: PCR amplification products of *PIK3R1* gene

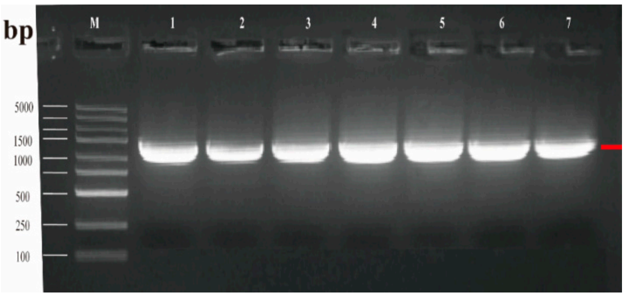

Supplementary Figure S3: Sanger sequencing peak of exon 7 DNA amplification fragment product of sheep PIK3R1 gene

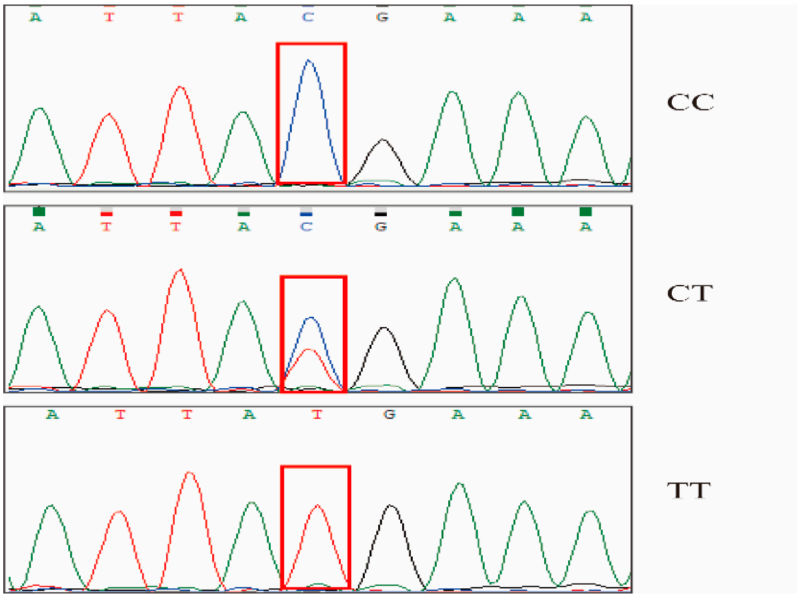

Supplement: Supplementary file 1 [file ijms-25-02951-s001.zip › ijms-2793180-supplementary.pdf]
